# Quality of life and return to work and sports after spinal ependymoma resection

**DOI:** 10.1038/s41598-022-09036-9

**Published:** 2022-03-23

**Authors:** Vicki M. Butenschoen, Till Gloßner, Isabel C. Hostettler, Bernhard Meyer, Maria Wostrack

**Affiliations:** 1 Department of Neurosurgery, School of Medicine, Klinikum Rechts Der Isar, Technical University Munich, Ismaningerstr. 22, 81675 Munich, Germany; 2 Department of Neurosurgery, Kantonspital St. Gallen, Rorschacher Strasse 95, 9007 St. Gallen, Switzerland

**Keywords:** Quality of life, CNS cancer, Chronic pain

## Abstract

Adult spinal ependymoma presents a rare low-grade tumor entity. Due to its incidence peak in the fourth decade of life, it mostly affects patients during a professionally and physically active time of life. We performed a retrospective monocentric study, including all patients operated upon for spinal ependymoma between 2009 and 2020. We prospectively collected data on professional reintegration, physical activities and quality-of-life parameters using EQ-5D and SF-36. Issues encountered were assessed using existing spinal-cord-specific questionnaires and free-text questions. In total, 65 of 114 patients agreed to participate. Most patients suffered from only mild pre- and postoperative impairment on the modified McCormick scale, but 67% confirmed difficulties performing physical activities in which they previously engaged due to pain, coordination problems and fear of injuries after a median follow-up of 5.4 years. We observed a shift from full- to part-time employment and patients unable to work, independently from tumor dignity, age and neurological function. Despite its benign nature and occurrence of formal only mild neurological deficits, patients described severe difficulties returning to their preoperative physical activity and profession. Clinical scores such as the McCormick grade and muscle strength may not reflect the entire self-perceived impairment appropriately.

## Introduction

Spinal cord ependymoma presents the most common spinal intradural neoplasm in adults and most often affects male patients in their forties^1–3^. Ependymomas usually show a non-aggressive growth pattern with a favorable outcome and are classified as WHO Grade 1 (myxopapillary, extramedullary) or 2 (mostly intramedullary) tumors^4^ while anaplastic ependymoma presents the least common subgroup of all ependymomas (WHO Grade 3 tumors)^5,6^. Due to their mostly slow-growing nature, gross total resection whilst preserving healthy surrounding tissue represents the first-line therapy, with promising 10-year, progression-free survival rates of 80–90%^7–9^. Tumor resection may be followed by a transient neurological deterioration, depending on tumor location, the presence of an accompanying syringomyelia^10^ or a surgical plane^11^ and the preoperative neurological status.

Compared to other benign neoplasms, the incidence of ependymomas peaks in the fourth to fifth decade of life, i.e., during a rather active and social life period. At this stage of life, even slight professional, or physical handicaps, such as reduced ability to work and participate in sport activities, may significantly impair quality of life in otherwise healthy adult patients^12–14^. As most published studies on spinal ependymoma focus on surgical technique, progression-free survival and quantifiable neurological signs, the real burden of disease may be underestimated regarding impairment in performance of daily life activities, such as sports and recreation due to subclinical deficits. Our aim was to investigate the actual impairment in patients who underwent spinal ependymoma resection, focusing on difficulties encountered in daily living activities and reintegration into work.

## Methods

### Patient cohort

We performed a retrospective assessment of all patients operated on for spinal ependymomas between January 2009 and September 2020 in our neurosurgical department in a tertiary care hospital. Patients’ medical records were retrospectively reviewed, and each patient was contacted individually by mail or telephone. Patient consent was required, and when obtained, a specific questionnaire was sent via mail with a stamped return envelope.

### Questionnaire

The questionnaire used in our study included an assessment of quality-of-life items using the European Quality of Life 5 Dimensions (EQ-5D) and the Short Form Health (SF-36).

We created a list of questions addressing education, occupational reintegration, and sport activities (with questions focusing on the pre- and postoperative situation, the frequency and the type of sports performed such as running, swimming, cycling or team sports). The questionnaires were validated for patients suffering from spinal cord injuries and modified according to previously conducted research^15^. At the end of the questionnaire, patients were able to answer free-text questions and add comments on difficulties encountered during their postoperative recovery.

### Health utility

We assessed patients’ health utility corresponding to a quantified number describing their health state or outcomes ranging between 0 (worst state imaginable) and 1 (perfect health) using the EQ-5D questionnaire. Utilities describe health state preference values and measure a perceived health status commonly used in health economics, health states were correlated with potential risk factors and neurological outcomes after surgery.

### Statistics

Statistical analyses were performed using SPSS Statistics 26 (IBM, Chicago, IL). Categorical data were compared using the chi-square test or Fisher’s exact test as appropriate. Mean values were compared using the independent samples *t* test.

The association between potential factors and reduced quality of life or sport activities was analyzed using an ANOVA. We assumed the following factors to be potentially predictive: presence of neurological deficits, perioperative complications, modified McCormick grade and age. To assess the correlation, we used Kendall’s tau correlation coefficient. All tests were two-sided; a *p* value < 0.05 was considered significant.

### Ethics and consent to participate

The presented study meets the ethical standards outlined in the Declaration of Helsinki, ethics approval was obtained by the local ethics committee (Prof. Dr. Georg Schmidt, Technical University Munich) and the positive vote was registered under the number 570/20 S. Written informed consent was obtained by all patients.

## Results

### Cohort population

**Figure 1:**
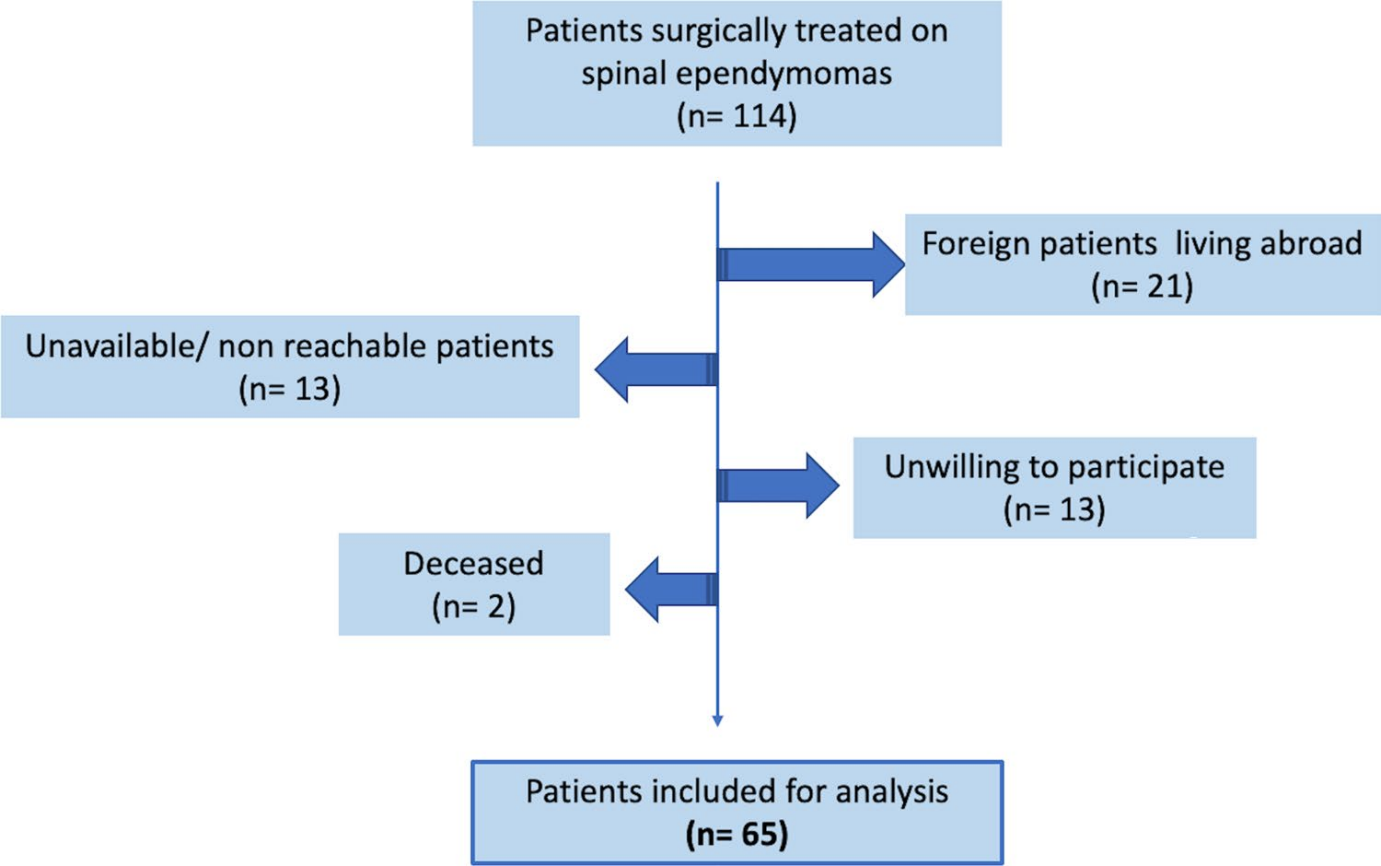
Flowchart describing the inclusion process of all patients assessed prospectively.

Overall, 114 consecutive patients were operated for spinal ependymomas between January 2009 and september 2020 in our department. We excluded 21 foreign patients living abroad due to missing follow-up reports. Thirteen patients declined participation (reasons included the personal aspect of the questions or non-willingness to participate in clinical trials), and 13 patients did not respond to our contact attempt. Two patients were deceased, leaving 65 patients included in the study assessment. Complete data and answered questionnaires were available for all included patients (flowchart, Fig. 1).

Median age was 45 years (IQ range 36 to 57 years), and most patients were female (34/65, 52.3%). All patients underwent surgery for spinal ependymomas: WHO°1 (19/65, 29.2%), WHO°2 (45/65, 69.2%) or WHO°3 (1/65, 1.5%), according to the WHO 2016 classification^16^. Multifocal lesions were diagnosed in 10/65 patients (15.4%), and four patients suffered from tumor recurrence (6.2%). Tumors were classified as intramedullary in 41.5% (27/65) and extramedullary in 58.5% of the cases (38/65) (Table 1).

**Table 1:** Demographics of the studied population including age at diagnosis, gender, location of the tumor, WHO° grading, time duration between surgery and follow-up questionnaires and presence of neurological deficits before surgery.

Age (years)	Median 45 years	IQ range: 36–57 years
Female sex	52.3%	
Intramedullary tumor	41.5%	
WHO		
1	29.2%	
2	69.2%	
3	1.5%	
Interval surgery-questionnaire	Median 5.4 years	IQ range: 3–8.5 years
Neurological deficit before surgery	43.1%	
Preoperative McCormick Grade		
1	75.4%	
2	20%	
3	4.6%	

Most patients underwent a unilateral approach for tumor resection (hemilaminectomy, foraminotomy or interlaminar fenestration in 41/65 patients 63.1%) or laminoplasty (20/65 patients, 30.8%) in one (38.5%, 25/65), two (26.2%, 17/65) or more segments (35.4%, 15/65). Mean surgery duration (LOS) was 183 min (IQ range 120–219 min), depending significantly on tumor location (extramedullary: mean 160 min, intramedullary: mean 215 min, *p* = 0.009). Neuromonitoring was used in most cases (56/65 patients, 85.2%). Most patients suffered from tumors at the lumbar (30/65, 46.2%) and thoracic (15/65, 23.1%) level. Cervical tumors were diagnosed in 13.8% of the cases, tumors of the cervicothoracic junction in 7.7% and ependymoma of the thoracolumbar junction in 6.2% of the patients. All patients underwent regular physiotherapy after surgery during the in-hospital stay.

### Neurological status

**Figure 2:**
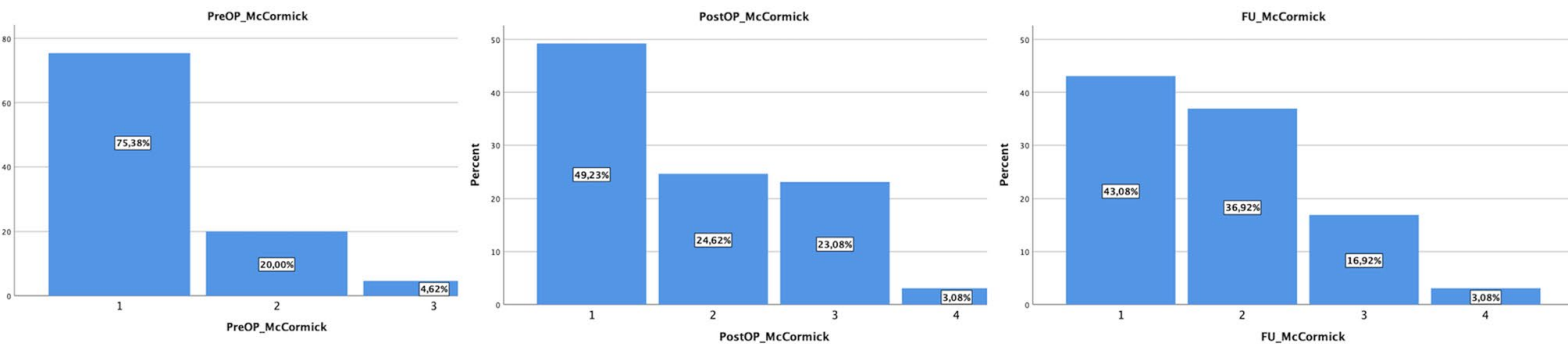
Preoperative, postoperative and follow-up McCormick grades describing clinical state and functional independency.

Preoperative neurological deficits were diagnosed in 28/65 patients (43.1%), including mostly mild sensory deficits (21/65, 32.3%), motor deficits (12/65, 18.5%, 9/12 patients only suffering from a mild impairment MRC 4/5, and 3/12 patients suffering from a MRC 3/5 paresis) and bladder dysfunction (4/65, 6.2%). Median preoperative modified McCormick grades showed only mild impairment: 75.4% had intact neurological function or, at most, minimal sensory deficits according to McCormick Grade I; 20% had mild neurological impairment, according to Grade II. Early postoperative deficits were observed in 37/65 patients (56.9%) with impaired sensory (31/65, 47.8%) and motor function (15/65, 23.1%, 3/15 with a new MRC 4/5 paresis, 10/15 patients deteriorating to a muscle strength MRC 3/5 paresis recovering until follow-up, 2/15 patients suffering from a severe mono- or paraparesis 1/5). Regarding median postoperative McCormick grades, Grade II accounted for 24.6%, and 23.1% of the patients were classified as McCormick Grade III after surgery. At follow-up, 52.3% of the patients described persistent neurological deficits, again most commonly affecting sensory function (47.7%), 80% of the patients were functionally independent (modified McCormick grade I or II; Fig. 2).

### Quality of life

The analyzed questionnaires included the EQ-5D assessment and SF-36 patient-reported survey. The assessment was performed after a median interval of 5.4 years after surgery (IQ range 3–8.5 years).

The mean utility (u) evaluated by the EQ-5D questionnaire was 0.676 (range 0–1), indicating a high burden of disease and self-perceived disability. Most patients were restrained in the pain category, demonstrating the lowest scores in this part of the questionnaire. Utility differed significantly between patients concerning difficulties returning to physical activities (u: 0.58 vs. 0.81, *p* = 0.03). We also found a strong dependence on sex: female patients demonstrated significantly lower levels of overall quality of life, with a mean u of 0.573 vs. 0.788 in male patients (*p* = 0.006, correlation coefficient 0.287). Health utilities were significantly lower in patients suffering from intramedullary ependymoma (u = 0.50) compared to patients operated on for extramedullary tumors (u = 0.80, *p* = 0.000). Furthermore, a correlation was found between the overall quality of life and the modified McCormick grade at follow-up (I: 0.828, II: 0.627, III: 0.432, *p* = 0.001, correlation coefficient − 0.447) as well as the presence of postoperative neurological deficits at follow-up (u: 0.554 vs. 0.809, *p* = 0.001, correlation coefficient − 0.417). Age did not affect health-related quality of life (*p* = 0.364).

Assessing the SF-36 general health questionnaires, the lowest scores were observed for vitality (mean 49.8%, range 10–95%) and role limitations due to physical constraints (mean 51.4%, range 0–100%). The highest scores were reported in the categories of social functioning (mean 72.9%, range 12.5–100%) and emotional role limitation (mean 67.7%, range 0–100%). Patients suffering from difficulties returning to sport activities after surgery had significantly lower scores in all categories on the SF-36 survey compared to patients denying any problems in returning to daily living (Table 2). Patients with intramedullary ependymoma scored significantly lower compared to patients with extramedullary ependymoma (*p* = 0.001 to 0.005 in all subgroups).

**Table 2:** Assessment of quality of life using the self-reported SF-36 survey depending on difficulties encountered performing sport activities.

Problem_Sport	% physical functioning	% role physical	% role emotional	% vitality	% mental health	% social functioning	% bodily pain	% general health	% score
**Yes**									
Mean	55.3**	33.5**	54.4**	42.6**	56.3**	63.8*	48.7**	46.1*	50.2**
Std. Deviation	31.6	38.7	41.4	20.4	18.9	30.0	25.3	25.0	19.0
Median	60	25	67	40	58	63	45	45	44
**No**									
Mean	82.5**	76.9**	85.9**	60.4**	76**	85.6*	78.5**	63.1*	73.9**
Std. Deviation	24.7	37.4	28.6	19.5	16.2	20.2	24.7	18.4	17.1
Median	90	100	100	65	76	94	85	63	78
**Total**									
Mean	66.3	51.2	67.2	49.8	64.3	72.7	60.8	53.0	59.8
Std. Deviation	31.8	43.5	39.6	21.8	20.3	28.4	28.9	23.9	21.6
Median	78	50	100	48	64	81	56	50	62
*p* value	0.000	0.000	0.001	0.001	0.000	0.002	0.000	0.004	0.000

Note the significant differences in scores in all categories for patients encountering problems in performing sport activities and returning to daily living activities.

*p < 0.005; **p < 0.001.

### Reintegration into professional employment

Assessing the pre- and postoperative occupations of our patients, we found a shift of full-time to part-time employment and patients who retired/were unable to work. Table 3 describes the findings focusing on pre- and postoperative education as well as pre- and postoperative occupation. Before surgery, most patients were full-time employees (34/65, 52.3%). After surgery, the amount of full-time employment diminished to 21/65 (32.5%), and the number of patients who retired early increased from 16.9 to 29.2%. Reasons for failure or difficulties encountered during reintegration were persistent pain (13/65, 20%), physical stress (11/65, 16.9%), impaired accessibility of work (1/65, 1.5%) and motor deficits in two cases (3.1%). Age, WHO grade, spinal level and sex did not significantly influence the ability to return to work (age *p* = 0.240, WHO grade *p* = 0.595, spinal level *p* = 0.102 and sex *p* = 0.621).

**Table 3:** Assessment of pre- and postoperative professional occupation.

	Occupation_preOP		Occupation_postOP		Changes
	Frequency	Percent	Frequency	Percent	
Student	1	1.5	1	1.5	0%
Full-time	**34**	**52.3**	21	32.3	− 20%
Part-time	9	13.8	11	16.9	+ 3%
Unemployed	3	4.6	3	4.6	0%
Housewife	2	3.1	1	1.5	− 1.6%
Retired	11	16.9	**19**	**29.2**	+ 12.3%
Unable to work	5	7.7	9	13.8	+ 6.1%
Total	65	100	65	100	

Note the shift from full-time employment to patients who retired and/or were unable to work (+ 12.3 and + 6.1%).

### Sports and daily living activities

**Figure 3:**
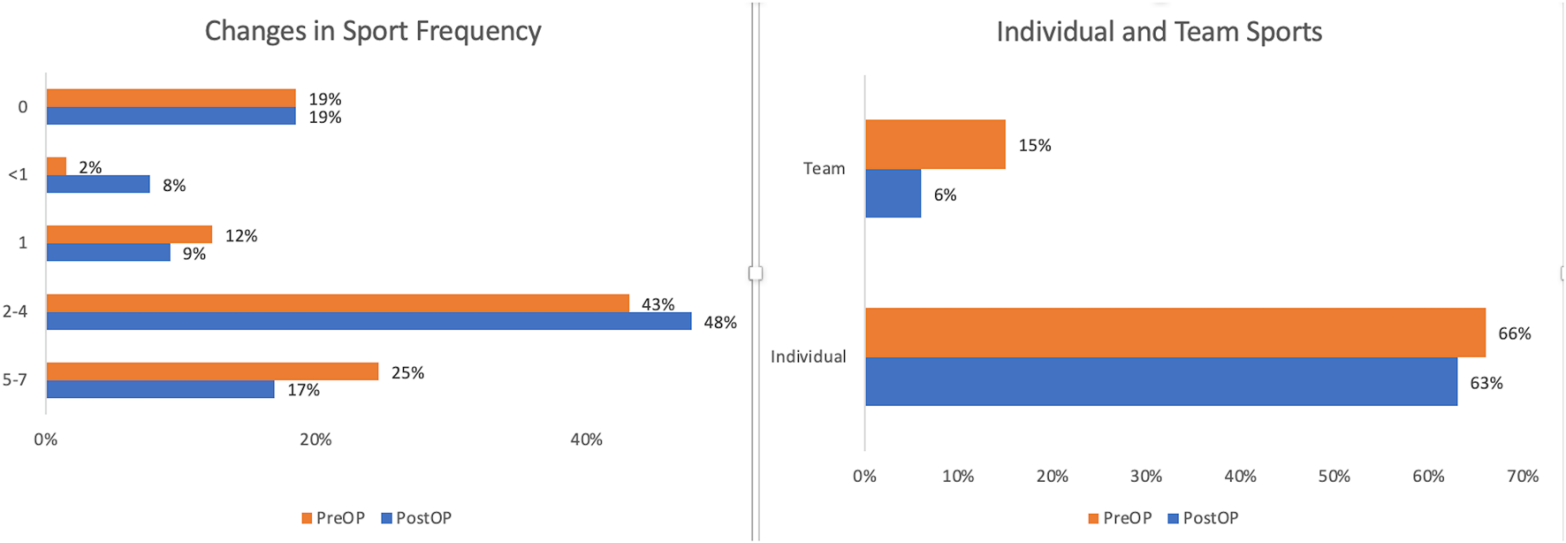
Changes of sport frequency and sport participation in team sports before and after surgery including the frequency of training (y-axis times/week) before and after surgery (left image) as well as the participation in individual and team sport activities (right image).

Before surgery, 66% of the patients performed individual sports and 15% trained and participated in team sports. After surgery, 63% continued to perform individual sports, but only 6% continued in team sports (Fig. 2). Assessing the frequency of sport activities, most patients attended sport activities 2 to 4 times/week before surgery, while 24.6% trained 5 to 7 times/week. After surgery, the amount of intensive training (5–7 times/week) diminished to 16.9% (11/65), while other patients remained active 2–4 times/week (47.7%, 31/65) (Fig. 3).

When asked if the encountered problems whilst performing sport activities related to their surgical treatment, 67.7% (44/65) of the patients confirmed pain (12/44, 27.3%), coordination problems (8/44, 18.2%), fear of injuries (5/44, 11.4%), motor deficits (9/44, 20.4%) and fatigue symptoms (5/44, 11.4%) to be related with the intervention. Following beneficial aspects were stated by 30 patients: increased mobility (15/65%, 23.1%), more health awareness (12/65, 18.5%) and social inclusion and participation (3/65, 4.6%).

### Prognostic factors

With regards to difficulties returning or performing sports related to medical issues, we found a significant association between occurrence of difficulties and preoperative as well as postoperative McCormick grades (*p* = 0.012 and *p* = 0.002, respectively), chosen approach (unilateral approach 56.2% vs. laminectomy 100% and laminoplasty 85%, *p* = 0.023) and number of operated segments (monosegmental approach 52% vs. 5 or more segments 100%, *p* = 0.022). Patients with postoperative neurological deficits described problems with sport activities in 69.4% (compared to 46.4% without neurological deficit, *p* = 0.063) and difficulties with professional reintegration in 48.6% (compared to 32.1%, *p* = 0.179).

Tumor dignity, sex and age did not affect participation in sport activities after surgery (*p* = 0.92, *p* = 0.994 and *p* = 0.510, respectively).

With regards to difficulties with professional reintegration, we found no significant association between early retirement or inability to work and tumor WHO grade, number of segments or sex (*p* = 0.595, *p* = 0.244 and *p* = 0.571, respectively). Patients reporting constraints returning to work tended to be younger (median age 42 years vs. 50 years, *p* = 0.056), but this finding reached only suggestive significance.

## Discussion

In our cohort, most patients experienced significant impairment in their daily living and changes in type and frequency of their sport activities after ependymoma resection, despite the benign nature of their tumor and overall mild formal neurological deficits. Even in cases with intact neurological function, patients reported a reduced health-related quality of life. The subclinical burden of disease in spinal ependymoma patients seems to be underestimated and health utility data showed a subjective health impairment comparable to glioma patients.

In our study, we assessed critical domains of daily quality of life in patients suffering from spinal ependymoma and evaluated specific utility scores. While most patients remained functionally independent according to the McCormick grading system, symptoms such as fatigue and depression were not reflected appropriately in our medical follow-up records and were only detected by specifically addressing issues encountered in daily living activities using our questionnaires. Fatigue has been described as occurring frequently in spinal ependymoma patients, affecting up to 52%^17^. The assessed utility score using the EQ-5D questionnaire was 0.676 in our cohort, which is somewhat even lower than previously reported values of patients suffering from intracranial malignant gliomas (0.70–0.80^18^)^19^. Interestingly, we found a significant difference in health-related quality of life between female and male patients, congruent with results presented in studies on quality of life in glioma patients, probably related to the greater dysfunction experienced by women at the same level of pain^20–22^. The high disease burden in spinal ependymoma patients may be explained by a disproportionately high impact of subclinical physical disability in an otherwise healthy young patient, who normally do not require adjuvant or disease-related in-hospital or outpatient therapy or medication, as well as by persistent pain symptoms as described by our patients.

The overall disease burden seems to be underestimated when only objectifiable clinical neurological status is included in the assessment of functional impairment. Disabilities in daily living activities are frequently encountered in cancer patients and widely described in the literature stating half of affected adults require assistance to perform activities of daily living^23^. Up until now, studies focus on malignant tumors and there has been no related data or evidence in benign spinal cord tumor patients. To our knowledge, we provide the first cohort population describing difficulties encountered in sports, recreation, and employment after surgery, as well as the first study assessing health utilities in this disease.

Over 67% of patients encountered difficulties when returning to sport activities due to pain, coordination problems, accessibility constraints and fatigue symptoms. While assessing encountered problems, we also evaluated positive reasons to return to sport activities. Patients described improved stability, social interaction, and improvement of pain symptoms with physical exercise. The potential rehabilitative effects of sport have been described in brain cancer patients and neuro-oncology in general^24,25^. A systematic review analyzing the potential benefit of rehabilitation in spinal cord tumor patients found positive effects on perceived quality of life and functionality^26^, and patients should be advised accordingly. Our study shows significant issues in accessing and performing sports in a population with benign tumors. These issues should be addressed and discussed with patients at follow-up appointments to improve accessibility of physical activity.

We did not compare our patient cohort to other tumor or spinal-trauma entities, as we only included patients undergoing spinal ependymoma surgery. While occupational reintegration data has been described for intracranial glioma patients^27,28^ and a small group of spinal ependymoma patients^13^, there is no evidence on sport activities and recreation in primary spinal cord tumor patients. Comparable available studies mostly included patients suffering from spinal cord injuries, with considerably worse neurological and clinical function and more possibilities for systematic reintegration and support programs^15,29^. While the benefits of physical activity in patients with spinal cord injuries have been extensively discussed and published^30^, we found only few relevant literature addressing physical activity and spinal rehabilitation in spinal intradural tumor patients. Patients in our cohort described impairment of coordination, sensation, fatigue and fear of injuries and accessibility problems, similar to patients suffering from spinal cord injuries, even when described as functionally independent. We therefore hypothesize that physical activity has a potential beneficial effect of in the neuro-oncological context of ependymoma patients. Unfortunately, patients in our department did not undergo a standardized rehabilitation program after surgery, and sports were not incorporated in their plan-of-care treatment. To date, screening programs are nonexistent, but explicit evaluation of daily life difficulties should be included during follow-up appointments to find room for improvement in a patient’s quality of life.

Regarding the ability to return to work, most patients were able to return to their profession after a stepwise reintegration. The timing of reintegration may vary depending on the physical demand of the profession and the clinical status of the patient.

Similar results were observed among patients suffering from cerebral gliomas^31^. In contrast to glioma studies, age did not significantly affect our patients’ abilities to resume their working lives, maybe due to their more homogeneous age group^32^. Age did not significantly affect the professional reintegration, therefore limiting a bias of potential age-related retirement.

### Limitations

We obtained data from patients in follow-up after surgery, without available preoperative scores. Our cohort might be subject to recall bias due to the time passed between our assessment and initial tumor resection. Also, memories about the preoperative state may have been subjectively disrupted. A prospective study can certainly reduce the occurrence of bias; however, data collection would take an inappropriately long time due to the low incidence of spinal ependymoma, making this option questionable. Our results may contain additional selection bias, since only 65 of 115 patients were included in the analysis. Furthermore, we unfortunately did not investigate possible solutions to improve participation in sports or help patients in their professional and occupational reintegration nor did we assess the actual time spent during each exercise session or the shift of different sports to other less demanding activities. Explicit identification and support of patients suffering from subjective daily living impairment should gain more importance at follow-up appointments and spinal rehabilitation discussed more commonly. Although we identified factors predicting impeded return to physical and leisure activities, we cannot offer modalities to improve reintegration based on our current results yet. Finally, we could not assess the socioeconomic burden of disease, as the time span of patients’ returning to their occupations was not investigated, neither did we investigate the influence of the COVID pandemic on the professional situation.

## Data Availability

The datasets used and/or analyzed during the current study are available from the corresponding author on reasonable request.

## References

[1] Ottenhausen M (2019). Intradural spinal tumors in adults-update on management and outcome. Neurosurg. Rev..

[2] Wostrack M (2018). Spinal ependymoma in adults: a multicenter investigation of surgical outcome and progression-free survival. J. Neurosurg. Spine.

[3] Brotchi J, Fischer G (1998). Spinal cord ependymomas. Neurosurg. Focus.

[4] Schwartz TH, McCormick PC (2000). Intramedullary ependymomas: clinical presentation, surgical treatment strategies and prognosis. J. Neurooncol..

[5] Lee JC (2019). Clinicopathologic features of anaplastic myxopapillary ependymomas. Brain Pathol..

[6] Celano E, Salehani A, Malcolm JG, Reinertsen E, Hadjipanayis CG (2016). Spinal cord ependymoma: A review of the literature and case series of ten patients. J. Neurooncol..

[7] Tarapore PE (2013). Pathology of spinal ependymomas: an institutional experience over 25 years in 134 patients. Neurosurgery.

[8] Lee SH (2013). Long-term outcomes of surgical resection with or without adjuvant radiation therapy for treatment of spinal ependymoma: a retrospective multicenter study by the Korea Spinal Oncology Research Group. Neuro Oncol..

[9] Bostrom A (2011). Surgery for spinal cord ependymomas: outcome and prognostic factors. Neurosurgery.

[10] Samii M, Klekamp J (1994). Surgical results of 100 intramedullary tumors in relation to accompanying syringomyelia. Neurosurgery.

[11] Garces-Ambrossi GL (2009). Factors associated with progression-free survival and long-term neurological outcome after resection of intramedullary spinal cord tumors: Analysis of 101 consecutive cases. J. Neurosurg. Spine.

[12] McGuire CS, Sainani KL, Fisher PG (2009). Incidence patterns for ependymoma: a surveillance, epidemiology, and end results study. J. Neurosurg..

[13] Behmanesh B (2020). Return to work and clinical outcome after surgical treatment and conservative management of patients with intramedullary spinal cord ependymoma. Sci. Rep..

[14] Armstrong TS, Vera-Bolanos E, Gilbert MR (2011). Clinical course of adult patients with ependymoma: Results of the Adult Ependymoma Outcomes Project. Cancer.

[15] Tasiemski T, Bergstrom E, Savic G, Gardner BP (2000). Sports, recreation and employment following spinal cord injury—A pilot study. Spinal Cord.

[16] Louis DN (2016). The 2016 World Health Organization classification of tumors of the central nervous system: A summary. Acta Neuropathol..

[17] Acquaye AA, Vera E, Gilbert MR, Armstrong TS (2017). Clinical presentation and outcomes for adult ependymoma patients. Cancer.

[18] Butenschoen VM, Kelm A, Meyer B, Krieg SM (2019). Quality-adjusted life years in glioma patients: A systematic review on currently available data and the lack of evidence-based utilities. J. Neurooncol..

[19] Drewes C, Sagberg LM, Jakola AS, Solheim O (2016). Quality of life in patients with intracranial tumors: Does tumor laterality matter?. J. Neurosurg..

[20] Mainio A, Hakko H, Niemela A, Koivukangas J, Rasanen P (2006). Gender difference in relation to depression and quality of life among patients with a primary brain tumor. Eur. Psychiatry.

[21] Janda M (2007). Quality of life among patients with a brain tumor and their carers. J. Psychosom. Res..

[22] Frick KD, Jones AS (2008). Gender bias in economic evaluation methods: Quality of life and family role effects. Womens Health Issues.

[23] Neo J, Fettes L, Gao W, Higginson IJ, Maddocks M (2017). Disability in activities of daily living among adults with cancer: A systematic review and meta-analysis. Cancer Treat Rev.

[24] Williams PT (2014). Reduced risk of brain cancer mortality from walking and running. Med. Sci. Sports Exerc..

[25] Cormie P, Nowak AK, Chambers SK, Galvao DA, Newton RU (2015). The potential role of exercise in neuro-oncology. Front. Oncol..

[26] Raj VS, Lofton L (2013). Rehabilitation and treatment of spinal cord tumors. J. Spinal Cord Med..

[27] Ryden I (2020). Return to work following diagnosis of low-grade glioma: A nationwide matched cohort study. Neurology.

[28] Starnoni D (2018). Returning to work after multimodal treatment in glioblastoma patients. Neurosurg. Focus.

[29] Tasiemski T, Kennedy P, Gardner BP, Taylor N (2005). The association of sports and physical recreation with life satisfaction in a community sample of people with spinal cord injuries. NeuroRehabilitation.

[30] McVeigh SA, Hitzig SL, Craven BC (2009). Influence of sport participation on community integration and quality of life: A comparison between sport participants and non-sport participants with spinal cord injury. J. Spinal Cord Med..

[31] Ng S, Herbet G, Moritz-Gasser S, Duffau H (2020). Return to work following surgery for incidental diffuse low-grade glioma: A prospective series with 74 patients. Neurosurgery.

[32] Senft C (2020). The ability to return to work: a patient-centered outcome parameter following glioma surgery. J. Neurooncol..

